# The neutrophil-to-lymphocyte ratio is associated with all-cause and cardiovascular mortality among individuals with hypertension

**DOI:** 10.1186/s12933-024-02191-5

**Published:** 2024-04-02

**Authors:** Xuexue Zhang, Rui Wei, Xujie Wang, Wantong Zhang, Mengxuan Li, Tian Ni, Weiliang Weng, Qiuyan Li

**Affiliations:** 1 Xiyuan Hospital, China Academy of Chinese Medical Sciences, Beijing, China; 2 China Academy of Chinese Medical Sciences, Beijing, China; 3 National Clinical Research Center for Chinese Medicine Cardiology, Xiyuan Hospital, China Academy of Chinese Medical Sciences, No. 1, Xiyuan Playground, Zhong Zhi Road, Hai Dian District, Beijing, 100091 China; 4 Department of General Medicine, Xiyuan Hospital, China Academy of Chinese Medical Sciences, No. 1, Xiyuan Playground, Zhong Zhi Road, Hai Dian District, Beijing, 100091 China

**Keywords:** All-cause mortality, Cardiovascular mortality, Hypertension, Neutrophil lymphocyte ratio

## Abstract

**Background:**

Identifying reliable prognostic markers is crucial for the effective management of hypertension. The neutrophil-to-lymphocyte ratio (NLR) has emerged as a potential inflammatory marker linked to cardiovascular outcomes. This study aims to investigate the association of NLR with all-cause and cardiovascular mortality among patients with hypertension.

**Methods:**

This study analyzed data from 3067 hypertensive adults in the National Health and Nutritional Examination Surveys (NHANES) from 2009 to 2014. Mortality details were obtained from the National Death Index (NDI). Restricted cubic spline (RCS) was deployed to visualize the association of the NLR with mortality risk. Weighted Cox proportional hazards models were employed to assess the independent association of NLR with mortality risk. Time-dependent receiver operating characteristic curve (ROC) analysis was conducted to access the predictive ability of NLR for survival. Mediation analysis was used to explore the indirect impact of NLR on mortality mediated through eGFR.

**Results:**

Over a median 92.0-months follow-up, 538 deaths occurred, including 114 cardiovascular deaths. RCS analysis revealed a positive association between NLR and both all-cause and cardiovascular mortality. Participants were stratified into higher (> 3.5) and lower (≤ 3.5) NLR groups. Weighted Cox proportional hazards models demonstrated that individuals with higher NLR had a significantly increased risk of all-cause (HR 1.96, 95% confidence interval (CI) 1.52–2.52, *p* < 0.0001) and cardiovascular mortality (HR 2.33, 95% CI 1.54–3.51, *p* < 0.0001). Stratified and interaction analysis confirmed the stability of the core results. Notably, eGFR partially mediated the association between NLR and both all-cause and cardiovascular mortality by a 5.4% and 4.7% proportion, respectively. Additionally, the areas under the curve (AUC) of the 3-, 5- and 10- year survival was 0.68, 0.65 and 0.64 for all-cause mortality and 0.68, 0.70 and 0.69 for cardiovascular mortality, respectively.

**Conclusion:**

Elevated NLR independently confers an increased risk for both all-cause and cardiovascular mortality in individuals with hypertension.

**Supplementary Information:**

The online version contains supplementary material available at 10.1186/s12933-024-02191-5.

## Introduction

Globally, hypertension stands out as the leading preventable cause of cardiovascular mortality and disease burden [1, 2]. Over the past three decades, the number of people aged 30–79 years with hypertension has doubled from 648 million in 1990 to 1278 million in 2019 [3]. Widely acknowledged as a major risk factor for stroke, cardiovascular diseases and kidney disease [4–6], hypertension is responsible for staggering 8.5 million deaths worldwide [3]. Unfortunately, a cross-sectional opportunistic study involving 89 countries revealed low-income countries face challenges in controlling hypertension compared to their wealthier counterparts [7]. Even in the United States, low-income groups bear a higher burden of hypertension and an elevated risk of cardiovascular events [8, 9]. To enhance the monitor of hypertension and related mortality, there is an imperative need to identify a prognostic parameter that is not only easily measurable but also cost-effective.

The neutrophil-to-lymphocyte ratio (NLR), derived from a complete blood count, serves as a crucial hematological parameter reflecting systemic inflammation and immune response [10]. It has garnered attention as a potential biomarker for various conditions, including cancer, inflammation, metabolic syndrome, affective, and neurodegenerative disorder [11–15]. Extensive research has consistently shown that an elevated NLR is positively linked to the incidence and prevalence of hypertension [16, 17] and various cardiovascular diseases [18]. A study based on data from the 1999–2014 National Health and Nutrition Examination Survey (NHANES), encompassing 32,454 individuals, highlighted the association of an increased NLR with all-cause mortality and mortality specifically attributed to heart disease, cerebrovascular disease and kidney disease [19]. However, the relationship between NLR and mortality risk in hypertensive individuals remains less explored. Therefore, this study aims to investigate the correlation between NLR and both all-cause and cardiovascular mortality in hypertensive patients through a population-based survey, which reflects the health status of adults across the United States.

## Materials and methods

### Data source

The data for this study was derived from NHANES database, a program conducted by the National Center for Health Statistics (NCHS) of the Centers for Disease Control and Prevention (CDC) [20]. NHANES is a nationally representative survey that collects information on the health and nutritional status of non-institutionalized civilian residents of the United States [21]. All participants provided informed consent before participating in this survey, and the NHANES dataset does not contain identifiable patient characteristics.

### Study population

Blood pressure measurements were performed by trained medical professionals using a mercury sphygmomanometer at mobile examination centers (MEC). Measurements were taken in a seated position, primarily using the right arm unless there were specific circumstances. After participants rested for 5 min, their blood pressure was measured three times consecutively, and the average of these three readings was used for subsequent analysis. Hypertension was defined as systolic blood pressure ≥ 140 mmHg, diastolic blood pressure ≥ 90 mmHg, or self-reported physician-diagnosed hypertension or the use of antihypertensive drugs [22]. Exclusions were applied to participants lacking complete survival data, blood cell counts, fasting sample weights, as well as those under 18 years old. Ultimately, a total of 3067 participants were included in this analysis, sourced from the 2009–2010 to 2013–2014 NHANES dataset (Additional file 1: Fig. S1).

### Measurement of NLR

Neutrophil and lymphocyte counts were determined by conducting a complete blood count on blood specimens using a Beckman Coulter automated blood analyzer in an MEC, expressed as × 10^3^ cells/µL. NLR was calculated as the absolute neutrophil count divided by the absolute lymphocyte count [23].

### Ascertainment of mortality and follow-up

Mortality status was determined by linking NHANES data with records from the National Death Index (NDI) available at https://www.cdc.gov/nchs/data-linkage/mortality-public.htm. Participants were categorized as deceased or alive based on information obtained from the NDI. Follow-up time was calculated from the date of the NHANES examination to the date of death or December 31, 2019, whichever occurred first. The International Classification of Diseases, Tenth Revision (ICD-10), was utilized to define the underlying causes of death. Cardiovascular mortality was specifically classified by the NCHS as deaths attributed to heart disease, identified by ICD-10 codes I00-I09, I11, I13, and I20-I51 [24].

### Assessment of covariates

The same set of covariates were considered for both all-cause and cardiovascular mortality analyses. Participant characteristics encompassed age, sex, race, education levels, smoking status, body mass index (BMI), diabetes, history of cardiovascular disease (CVD), HbA1c, high-density lipoprotein cholesterol (HDL), low-density lipoprotein cholesterol (LDL), total cholesterol (TC), triglyceride (TG), and estimated glomerular filtration rate (eGFR). Race was categorized as non-Hispanic white, non-Hispanic black, Mexican American, and others. Education levels were grouped as “less than high school”, “high school or general educational development (GED)”, or “more than high school” [25]. Smoking was categorized as never smoking (having smoked < 100 cigarettes in life), former smoking (having smoked ≥ 100 cigarettes in life but not smoking currently), and current smoking (smoked cigarettes every day or some days at the time of the survey) [26]. BMI was calculated as weight in kilograms divided by the square of height in meters and classified as normal (< 25.0 kg/m^2^), overweight (25.0 to 30 kg/m^2^), and obese (≥ 30.0 kg/m^2^) [27]. The definition of diabetes mellitus relied on affirmative responses to any of the following questions: “Have you ever been told by a doctor that you have diabetes?” or “Are you now taking insulin?” or “Are you now taking diabetes pills to lower your blood sugar?” [28]. Cardiovascular disease included coronary heart disease, heart failure, myocardial infarction, and stroke. The CKD Epidemiology Collaboration (CKD-EPI) equation was employed to estimate calculated eGFR [29].

### Statistical analysis

This analysis took into account the complex NHANES sample design by considering appropriate sample weights, stratifications, and clustering. Sample weights for analysis were calculated as fasting sample 2-year MEC weight divided by 3. Categorical variables were compared using the survey-weighted Chi-square test, while continuous variables were compared using the survey-weighted linear regression.

Maximally selected rank statistics, facilitated by the 'maxstat' package, were employed to discern the optimal cutoff point for NLR, which segregated participants into lower and higher NLR groups [30]. To explore potential non-linear relationships between NLR and all-cause mortality, as well as cardiovascular mortality among individuals with hypertension, restricted cubic splines (RCS) were deployed. Various knot placements between 3 and 7 were tested, with the model featuring the lowest Akaike Information Criterion value selected for RCS, ultimately utilizing 3 knots. The inflection point was determined based on the shape of the RCS.

Survey-weighted Cox proportional hazards models were employed to assess the independent association of NLR with all-cause and cardiovascular mortality among individuals with hypertension. Outcomes were presented as model 1, model 2 (adjusted for age, sex, race, BMI, education level, and smoking status), and model 3 (adjusted for age, sex, race, BMI, smoking status, education level, diabetes, history of CVD, HDL, LDL, TG, TC, HbA1c, and eGFR).

Survival analysis, conducted using the Kaplan–Meier method, evaluated the survival probability of different groups of individuals with hypertension based on NLR levels, with comparison using the log-rank test. Stratified and interaction analyses were performed considering variables such as age (< 65 and ≥ 65 years old), sex (Male/Female), smoking status (never and former/current), BMI (< 25, 25–30, and ≥ 30 kg/m^2^), diabetes (Yes/No) and history of CVD (Yes/No).

The ‘timeROC’ package was employed to evaluate the accuracy of NLR in predicting survival outcomes at various time points [31]. A mediation analysis was carried out to access the indirect impact of NLR on mortality mediated through eGFR. Statistical analyses were conducted using R and EmpowerStats Software. A 2-sided *p* value less than 0.05 was deemed statistically significant.

## Results

### Baseline characteristics of study participants

A total of 3067 participants were eligible for the current study (Additional file 1: Fig. S1). The optimal NLR cutoff value for survival was 3.5, leading to the categorization of participants into two groups: a higher NLR group (NLR > 3.5, n = 381) and a lower NLR group (NLR ≤ 3.5, n = 2686) (Additional file 1: Fig. S2). Compared to the lower NLR group, individuals in the higher NLR group demonstrated several notable differences. They tended to be older, with a larger proportion of white race, having diabetics, and a history of CVD. The higher NLR group also had a smaller proportion of people who never smoked and exhibited lower TC, TG, LDL, lymphocytes and eGFR (Table 1).

**Table 1:** Baseline characteristics of included participants

Characteristics	Total (n = 3067)	Lower NLR (n = 2686)	Higher NLR (n = 381)	P-value
Age, years	57.1 (56.4, 57.7)	56.3 (55.6, 56.9)	62.5 (60.8, 64.3)	< 0.0001
Sex, %				0.8896
Male	48.4 (46.5, 50.3)	48.3 (46.1, 50.6)	48.9 (42.1, 55.6)	
Female	51.6 (49.7, 53.5)	51.7 (49.4, 53.9)	51.1 (44.4, 57.9)	
Race, %				< 0.0001
Non-Hispanic White	69.6 (64.8, 74.0)	67.9 (63.0, 72.4)	81.4 (75.1, 86.3)	
Non-Hispanic Black	14.5 (11.9, 17.5)	15.8 (13.0, 19.1)	5.2 (3.4, 8.0)	
Mexican American	5.6 (4.0, 7.8)	5.9 (4.2, 8.1)	3.8 (2.1, 6.6)	
Others	10.3 (8.6, 12.3)	10.4 (8.6, 12.5)	9.7 (6.7, 13.7)	
Education levels, %				0.9317
Less than high school level	6.7 (5.7, 7.8)	6.7 (5.7, 8.0)	6.2 (4.2, 9.1)	
High school or equivalent	37.2 (34.3, 40.3)	37.3 (34.2, 40.5)	36.9 (30.7, 43.5)	
Greater than high school level	56.1 (53.1, 59.1)	56.0 (52.7, 59.2)	56.9 (50.7, 63.0)	
Not recorded	0.0 (0.0, 0.1)	0.0 (0.0, 0.1)	0.1 (0.0, 0.4)	
Smoking status, %				0.0216
Never	49.4 (46.7, 52.1)	50.5 (47.7, 53.2)	41.9 (35.7, 48.5)	
Former	32.5 (29.9, 35.2)	31.3 (28.7, 34.0)	40.7 (33.0, 48.8)	
Current	18.1 (16.2, 20.2)	18.2 (16.3, 20.3)	17.4 (12.0, 24.5)	
Not recorded	0.3 (0.2, 0.6)	0.4 (0.2, 0.7)	0.0 (0.0, 0.0)	
BMI, kg/m^2^	31.3 (30.9, 31.7)	31.4 (31.0, 31.8)	30.7 (29.5, 31.9)	0.246
BMI category, %				0.0009
< 25	17.9 (16.1, 19.8)	16.9 (15.3, 18.7)	24.6 (19.3, 30.8)	
25–30	31.4 (29.5, 33.4)	31.8 (29.6, 34.0)	29.2 (23.4, 35.7)	
≥ 30	50.7 (48.3, 53.0)	51.3 (49.0, 53.6)	46.2 (38.9, 53.7)	
Not recorded	1.1 (0.7, 1.6)	0.9 (0.6, 1.4)	2.4 (1.4, 4.2)	
Diabetes, %				0.0334
No	81.0 (79.0, 82.9)	81.8 (79.5, 83.9)	75.6 (69.5, 80.7)	
Yes	19.0 (17.1, 21.0)	18.2 (16.1, 20.5)	24.4 (19.3, 30.5)	
Not recorded	0.0 (0.0, 0.2)	0.0 (0.0, 0.2)	0.0 (0.0, 0.0)	
History of CVD, %				< 0.0001
No	82.6 (80.9, 84.2)	84.7 (83.2, 86.1)	68.2 (61.2, 74.5)	
Yes	17.4 (15.8, 19.1)	15.3 (13.9, 16.8)	31.8 (25.5, 38.8)	
Not recorded	0.4 (0.2, 0.7)	0.4 (0.3, 0.8)	0.1 (0.0, 1.0)	
HbA1c, %	5.9 (5.9, 6.0)	5.9 (5.8, 6.0)	6.0 (5.8, 6.1)	0.4054
HDL, mg/dl	52.5 (51.6, 53.4)	52.6 (51.6, 53.5)	51.6 (49.6, 53.6)	0.3599
TC, mg/dl	195.2 (193.1, 197.4)	196.7 (194.2,199.1)	185.3 (179.3, 191.2)	0.0016
TG, mg/dl	143.0 (136.8, 149.1)	144.8 (137.8, 151.7)	130.6 (122.8, 138.5)	0.0088
LDL, mg/dl	114.7 (112.9, 116.4)	115.7 (113.9, 117.6)	107.4 (102.0, 112.8)	0.0072
SCR, mg/dl	1.0 (0.9, 1.0)	0.9 (0.9, 0.9)	1.1 (1.0, 1.3)	0.0025
Lymphocyte, × 10^9^/L	2.0 (1.9, 2.0)	2.1 (2.0, 2.1)	1.3 (1.3, 1.4)	< 0.0001
Neutrophil, × 10^9^/L	4.2 (4.1, 4.3)	3.9 (3.8, 4.0)	6.2 (5.9, 6.5)	< 0.0001
eGFR, mL/min/1.73m^2^	83.4 (82.3, 84.5)	84.5 (83.2, 85.8)	75.7 (72.8, 78.6)	< 0.0001

BMI: body mass index; CVD: cardiovascular disease; HDL: high-density lipoprotein cholesterol; LDL: low-density lipoprotein cholesterol; TC: total cholesterol; TG: triglyceride; SCR: serum creatinine; eGFR: estimated glomerular filtration rate. Continuous variables are presented as the mean and 95% confidence interval, category variables are described as the percentage and 95% confidence interval. Continuous variables are presented as the mean and 95% confidence interval, category variables are described as the percentage and 95% confidence interval

### Associations between NLR and all‑cause mortality in patients with hypertension

**Fig. 1:**
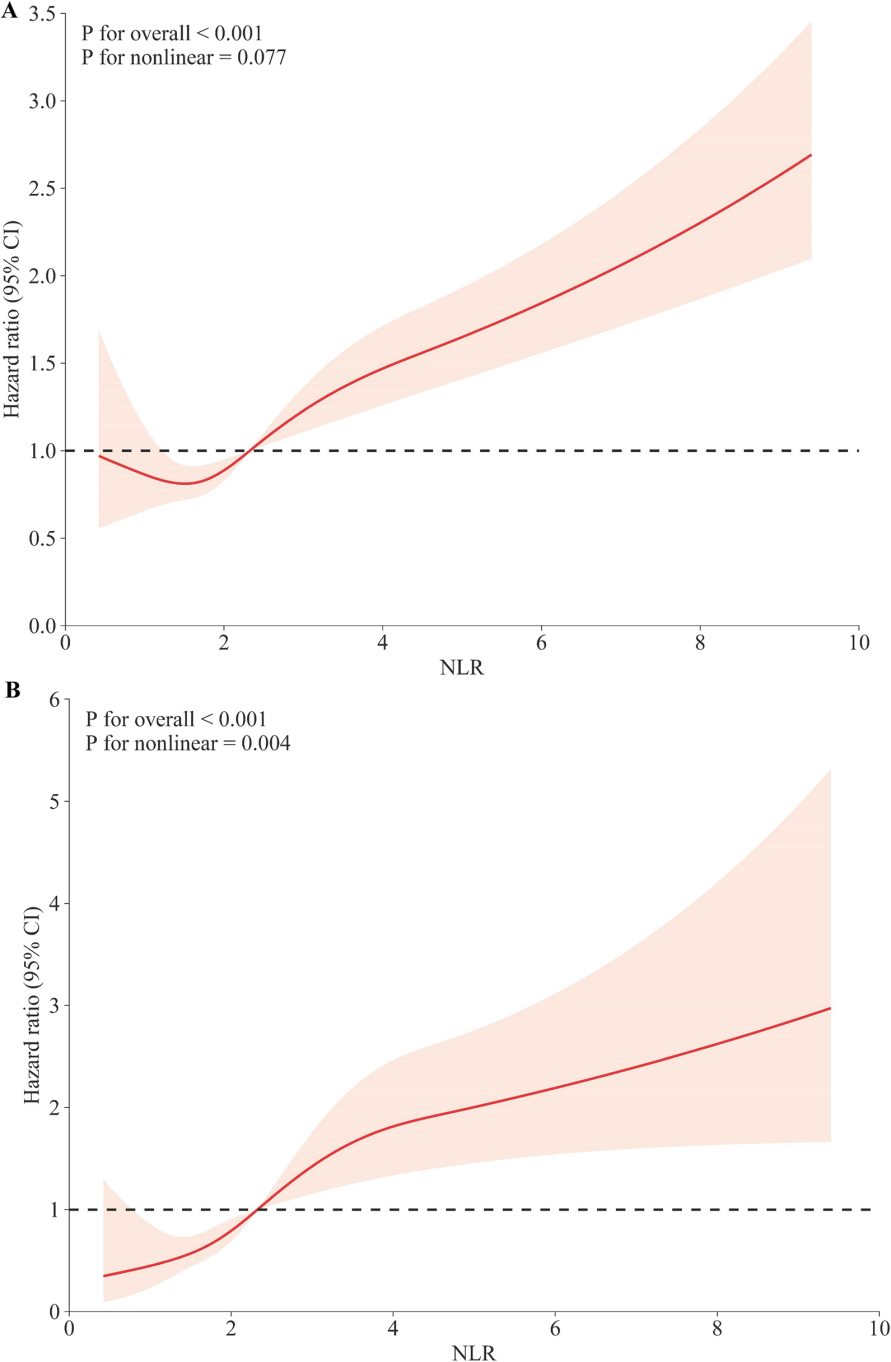
The association of NLR with all-cause (**A**) and cardiovascular mortality (**B**) among hypertension visualized by restricted cubic spline. Hazard ratios were adjusted for age, sex, race, BMI, smoking status, education level, diabetes, history of CVD, HDL, LDL, TG, TC, HbA1c, and eGFR

During a median follow-up period of 92.0 months (interquartile range (IQR), 72.0–111.0 months), 538 (17.5%) of the 3067 participants died, with 144 (4.7%) deaths attributed to cardiovascular disease. RCS analysis revealed a positive linear association between NLR and all-cause mortality (*p* for nonlinear = 0.077) (Fig. 1A). In model 1, the risk of all-cause mortality significantly increased with rising NLR values (HR 1.19, 95% confidence interval (CI) 1.13–1.24, *p* < 0.0001) (Table 2). After multivariate adjustment, each one-unit increase in NLR was associated with a 13% (Model 2, HR 1.13, 95% CI 1.08–1.18,* p* < 0.0001) and 12% (Model 3, HR 1.12, 95% CI 1.07–1.17, *p* < 0.0001) (Table 2) increased risk of all-cause mortality, respectively.

**Table 2:** The relationships between NLR and mortality in hypertension

Characteristic	Model 1		Model 2		Model 3	
	HR (95% CI)	P value	HR (95% CI)	P value	HR (95% CI)	P value
All-cause mortality						
NLR	1.19 (1.13, 1.24)	< 0.0001	1.13 (1.08, 1.18)	< 0.0001	1.12 (1.07, 1.17)	< 0.0001
NLR category						
Lower NLR	Ref		Ref		Ref	
Higher NLR	3.35 (2.79, 4.02)	< 0.0001	2.26 (1.81, 2.82)	< 0.0001	1.96 (1.52, 2.52)	< 0.0001
Cardiovascular mortality						
NLR	1.19 (1.13, 1.26)	< 0.0001	1.15 (1.08, 1.22)	< 0.0001	1.15 (1.08, 1.23)	< 0.0001
NLR category						
Lower NLR	Ref		Ref		Ref	
Higher NLR	3.92 (2.79, 5.51)	< 0.0001	3.04 (2.07, 4.47)	< 0.0001	2.33 (1.54, 3.51)	< 0.0001

Model 1, unadjusted; Model 2, adjusted for age, sex, race, BMI, education level, and smoking status; Model 3, adjusted for age, sex, race, BMI, smoking status, education level, diabetes, history of CVD, HDL, LDL, TG, TC, HbA1c, and eGFR

**Fig. 2:**
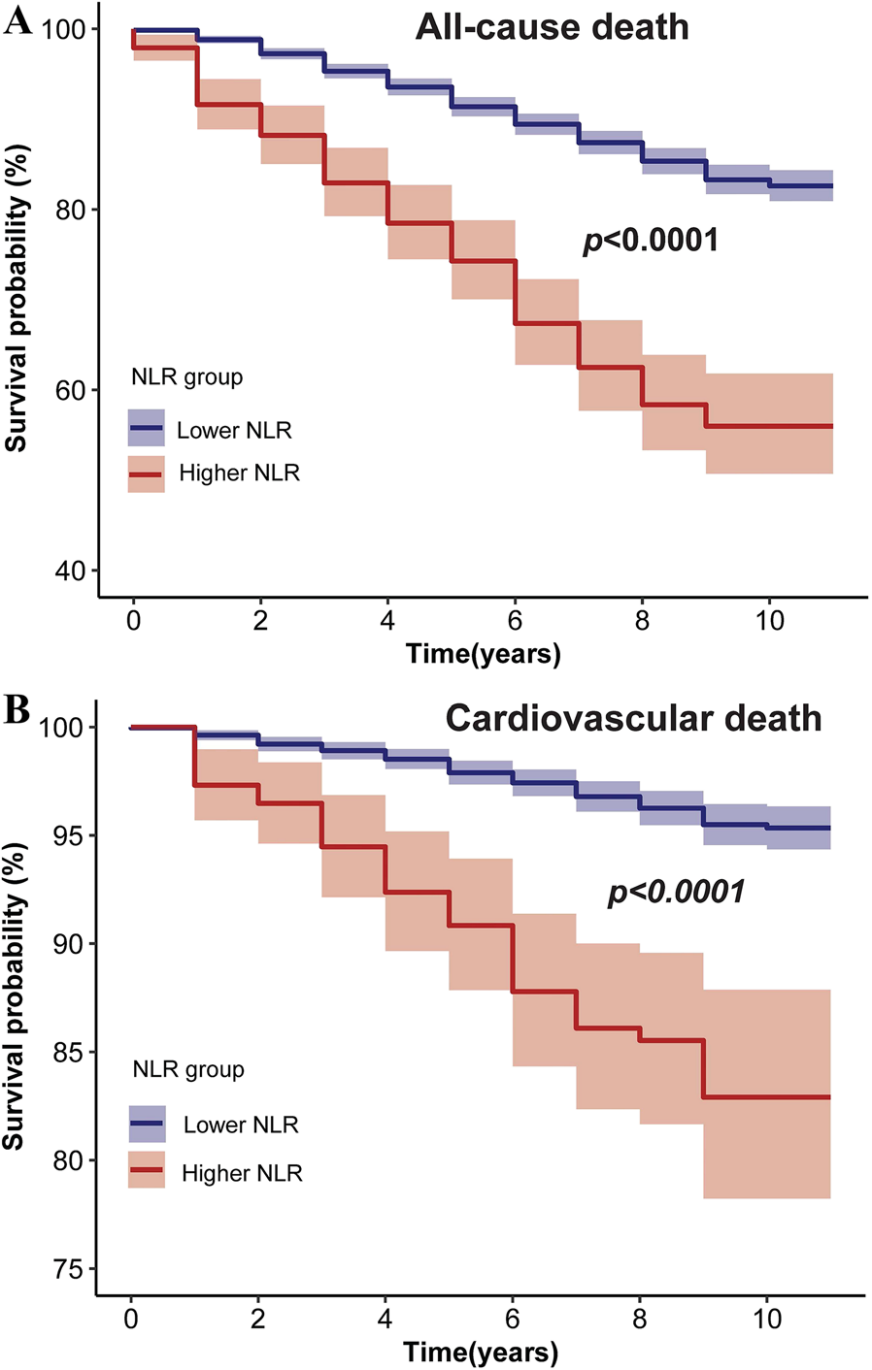
Kaplan–Meier curves of the survival rate with higher (> 3.5) and lower (≤ 3.5) NLR values. **A** All-cause mortality; **B** cardiovascular mortality

Survival curve analysis showed a significant decrease in the survival rate in the higher NLR group compared to the lower NLR group (*p* < 0.0001) (Fig. 2A). Cox regression analysis demonstrated a substantial increase in all-cause mortality in the higher NLR group, from model 1(HR 3.35, 95% CI 2.79–4.02, *p* < 0.0001) to Model 2 (HR 2.26, 95% CI 1.81–2.82, *p* < 0.0001) and model 3 (HR 1.96, 95% CI 1.52–2.52, *p* < 0.0001), compared with lower NLR group (Table 2).

Subgroup analysis was conducted to investigate the relationship between NLR and all-cause mortality, based on age, sex, smoking status, BMI, diabetes and history of CVD. The results revealed a consistent correlation, with no significant interaction observed between these characteristics and NLR (*p* for interaction > 0.05) (Table 3).

**Table 3:** Subgroup analysis of the associations between NLR and mortality among hypertension

Characteristics	All-cause mortality			P interaction	Cardiovascular mortality			P interaction
	Lower NLR	Higher NLR			Lower NLR	Higher NLR		
		HR (95% CI)	P value			HR (95% CI)	P value	
Age				0.3528				0.6377
< 65	Ref	2.45 (1.51,3.96)	0.0003		Ref	3.34 (1.24, 8.99)	0.0172	
≥ 65	Ref	1.89 (1.50, 2.39)	< 0.0001		Ref	2.61 (1.69, 4.02)	< 0.0001	
Sex				0.7958				0.5632
Male	Ref	1.87 (1.4,2.44)	< 0.0001		Ref	3.21 (1.94,5.33)	< 0.0001	
Female	Ref	1.96 (1.40, 2.76)	< 0.0001		Ref	3.21 (1.94,5.33)	< 0.0001	
Smoking status				0.6255				0.4922
Never	Ref	1.99 (1.43,2.76)	< 0.0001		Ref	2.33 (1.34,4.06)	0.0028	
Former/current	Ref	1.84 (1.40,2.41)	< 0.0001		Ref	3.17 (1.82,5.52)	< 0.0001	
BMI				0.7612				0.7453
< 25	Ref	1.84 (1.40,2.41)	< 0.0001		Ref	3.56 (1.45,8.70)	0.0054	
25–30	Ref	1.99 (1.36,2.92)	0.0004		Ref	3.56 (1.45,8.70)	0.0054	
≥ 30	Ref	1.67 (1.18,2.37)	0.0039		Ref	2.51 (1.38,4.55)	0.0024	
Diabetes				0.1582				0.1575
No	Ref	1.72 (1.33,2.23)	< 0.0001		Ref	2.26 (1.35,3.78)	0.0019	
Yes	Ref	2.39 (1.66,3.45)	< 0.0001		Ref	4.08 (2.18,7.64)	< 0.0001	
History of CVD				0.3115				0.1799
No	Ref	1.78 (1.34,2.38)	< 0.0001		Ref	4.08 (2.18,7.64)	< 0.0001	
Yes	Ref	2.25 (1.64,3.07)	< 0.0001		Ref	3.54 (2.13,5.88)	< 0.0001	

HRs were adjusted for age, sex, race, BMI, smoking status, education level, diabetes, history of CVD, HDL, LDL, TG, TC, HbA1c, and eGFR

### Associations between NLR and cardiovascular mortality in patients with hypertension

RCS analysis revealed a positive nonlinear association between NLR and cardiovascular mortality (*p* for nonlinear = 0.004) (Fig. 1B). Segmented Cox regression analysis pinpointed an inflection point at 2.3 (Additional file 1: Table S1). In model 1, the risk for cardiovascular mortality significantly increased with higher NLR values (HR 1.19, 95% CI 1.13–1.26, *p* < 0.0001) (Table 2). After comprehensive adjustment, each one-unit increase in the NLR value was associated with a 15% elevation in the risk of cardiovascular mortality (Model 3, HR 1.15, 95% CI 1.08–1.23, *p* < 0.0001) (Table 2).

Survival curve analysis also showed a significant decrease in survival rate in the higher NLR group compared to the lower NLR group (*p* < 0.0001) (Fig. 2B). Cox regression analysis confirmed a substantial increase in cardiovascular mortality in the higher NLR group, from model 1 (HR 3.92, 95% CI 2.79–5.51, *p* < 0.0001) to Model 2 (HR 3.04, 95% CI 2.07–4.47, *p* < 0.0001) and model 3 (HR 2.33, 95% CI 1.54–3.51, *p* < 0.0001), compared with the lower NLR group (Table 2).

Subgroup analysis explored the relationship between NLR and cardiovascular mortality, based on age, sex, smoking status, BMI, diabetes and history of CVD. The core results remained consistent, and there was no significant interaction between these characteristics and NLR (*p* for interaction > 0.05) (Table 3).

### The predictive ability of NLR for all‑cause and cardiovascular mortality in patients with hypertension

**Fig. 3:**
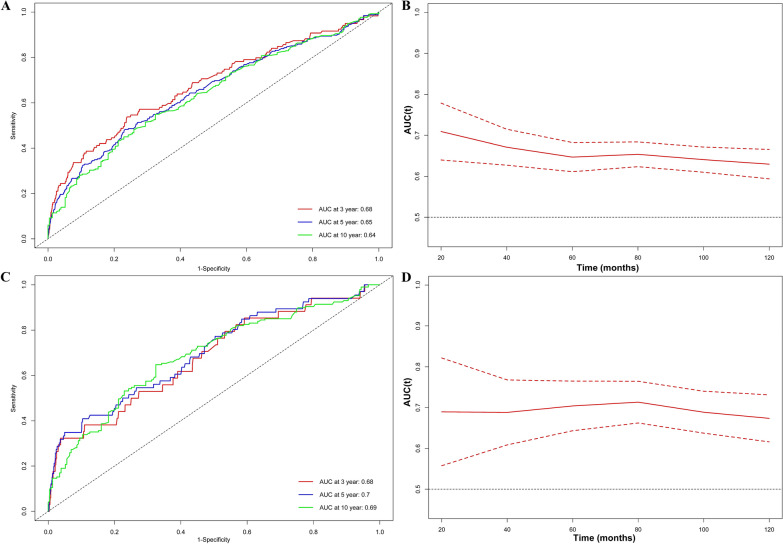
Time-dependent ROC curves and time-dependent AUC values (with 95% confidence band) of the NLR for predicting all-cause mortality (**A**, **B**) and cardiovascular mortality (**C**, **D**)

Time-dependent receiver operating characteristic curve (ROC) analysis showed the area under the curve (AUC) of the NLR was 0.68, 0.65 and 0.64 for 3-year, 5-year and 10-year all-cause mortality, respectively (Fig. 3A and B). Also, the AUC of the NLR was 0.68, 0.70 and 0.69 for 3-year, 5-year and 10-year cardiovascular mortality, respectively (Fig. 3C and D). This indicates that NLR possesses similarly effective predictive ability for mortality across different time periods. In addition, we calculated the predictive ability of using lymphocytes and neutrophils alone for all-cause and cardiovascular mortality in hypertensive patients. The results indicate that, whether over 3 years, 5 years, or 10 years, the predictive ability of using lymphocytes and neutrophils alone for mortality is inferior to that of NLR (Additional file 1: Fig. S3).

### Mediation analysis of NLR for all‑cause and cardiovascular mortality in patients with hypertension

**Fig. 4:**
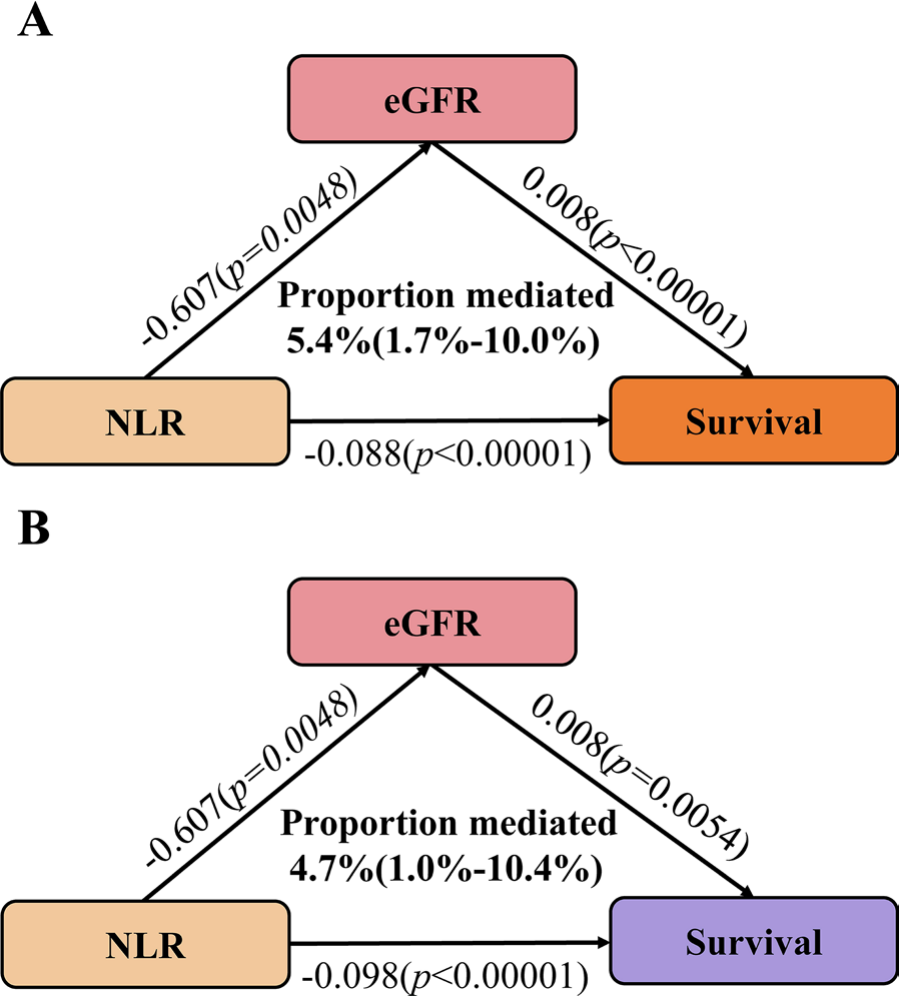
The mediating effect of eGFR on the relationship between NLR and survival (**A**, all-cause death; **B**, cardiovascular death). Adjusted for age, sex, race, BMI, smoking status, education level, diabetes, history of CVD, HDL, LDL, TG, TC, and HbA1c

Mediation analysis explored the mediating effect of eGFR on the relationship between NLR and both all-cause and cardiovascular mortality. Specifically, NLR was negatively correlated with eGFR (β ± SE = − 0.607 ± 0.215, *p* = 0.0048), while eGFR was positively correlated with survival for all-cause (β ± SE = 0.008 ± 0.002, *p* < 0.00001) and cardiovascular cause (β ± SE = 0.008 ± 0.003,* p* = 0.0054). Ultimately, 5.4% (95% CI 1.7% -10.0%) and 4.7% (95% CI 1.0%–10.4%) of the observational association of NLR with risk of all-cause and cardiovascular mortality was mediated through eGFR (Fig. 4A and B).

## Discussion

This study comprehensively analyzed the correlation between NLR and all-cause as well as cardiovascular mortality using multiple methods among hypertensive populations. Through the analysis of physical examination data from 3067 hypertensive participants in the NHANES, we found a positive correlation between NLR and both all-cause mortality and cardiovascular mortality. Furthermore, NLR demonstrated an effective predictive ability for both types of mortality over 3, 5, and 10 years, compared to lymphocytes and neutrophils alone. Mediation analyses showed that eGFR played a mediating role in the relationship between NLR and mortality. These findings remained consistent across various sensitivity and stratified analyses.

Neutrophil count and lymphocyte count, routinely assessed in blood tests due to their simplicity and affordability, collectively provide valuable insights into systemic inflammatory status and the balance between natural immunity (neutrophils) and acquired immunity (lymphocytes). The ratio of these counts, known as the NLR, emerges as a more predictive indicator than either parameter alone [18]. Recent studies have underscored the pivotal role of inflammation and immune responses in the onset and persistence of hypertension, contributing to elevate blood pressure by triggering vascular inflammation and microvascular remodeling [32]. Furthermore, NLR has demonstrated a predictive ability concerning the severity and mortality of cardiovascular diseases, encompassing acute coronary syndrome, coronary heart disease, and heart failure [33, 34]. In addition, an elevated NLR has been associated with increased mortality in chronic lower respiratory diseases, pneumonia, sepsis, kidney disease and cancer [19, 33]. The above evidences suggest that NLR may potentially evolve into a prognostic marker for disease progression and mortality risk in different populations. However, the potential of NLR remains unclear within the hypertensive population.

Our study aligns with the prior research of Xu et al. [17], indicating a higher proportion of hypertensive patients in the higher NLR group with characteristics such as advanced age, non-Hispanic white, diabetes, CVD history and lower eGFR (Table 1). This underscores the importance of managing comorbidities, including diabetes, cardiovascular disease and chronic kidney disease, alongside the prevention and treatment of hypertension. Our research results showed significant disparities in all-cause mortality and cardiovascular mortality between the higher and lower NLR groups when NLR was used as a categorical variable (Fig. 2 and Table 2). Additionally, using NLR values as continuous variables demonstrated their predictive ability for the risk of all-cause mortality and cardiovascular mortality over the next 3, 5 and 10 years (Fig. 3). This suggests that neutrophils and lymphocytes, as crucial components, play a role in chronic inflammation and immune response throughout the entire process of hypertension, consistent with previous research by Siedlinski et al. [35].

Numerous studies consistently demonstrated that, beyond its role in cardiovascular disease, low-grade chronic inflammation contributes to various complications associated with hypertension, including kidney diseases, retinopathy and neurodegenerative diseases [36–38]. Remarkably, during our mediation analysis, we found that NLR increased the risk of all-cause mortality and cardiovascular mortality partially by lowering eGFR, which revealed the potential underlying mechanisms (Fig. 4). Renal dysfunction is intricately linked to hypertension, with factors such as increased renal sympathetic nervous activity (RSNA), elevated levels of antidiuretic hormones or relatively insufficient levels of natriuretic hormones contributing to elevated blood pressure [36]. The observed decreased eGFR among hypertensive individuals in the high NLR group provides insights for investigating its potential mechanism. Compared to inflammatory and immune-mediated kidney diseases such as diabetic kidney disease and immunoglobulin A nephropathy [39, 40], which are also associated with elevated NLR, we propose a similar mechanistic basis for renal pathology in hypertensive individuals. A higher NLR suggests a diffuse proliferative pattern of injury characterized by endocapillary hypercellularity and leukocyte infiltration, including neutrophils [41]. Among these, neutrophils release proteases, arachidonic acid, and oxygen free radicals, contributing to the degradation of the glomerular basement membrane, glomerular sclerosis, renal fibrosis, and tubular damage, ultimately leading to a decline in eGFR [39]. Conversely, lymphocytes serve as regulatory and protective components of inflammatory mediators [42]. A decrease in eGFR not only signifies renal dysfunction but also indicates pathological conditions, including inflammation, microvascular disorders, and heightened oxidative stress, which are common progressive pathways observed in cardiovascular disease and chronic kidney disease, culminating in all-cause mortality [43]. Furthermore, a reduced eGFR can impact the volume and capacitance of circulation, resulting in vascular medial calcification, valvular calcification, and left ventricular hypertrophy, thereby increasing cardiovascular mortality [44]. The aforementioned pathological response in hypertensive individuals contributes to progressive kidney damage, leading to an escalation in mortality and rendering hypertension more resistant to treatment. A recent study has indicated that employing interleukin-1β inhibition in individuals with a prior myocardial infarction and a high-sensitivity C-reactive protein level ≥ 2 mg/L may lead to a reduction in cardiovascular adverse events without lowering blood pressure [45]. Therefore, it is reasonable to speculate that in hypertensive patients with elevated NLR, the combined use of antihypertensive medications and anti-inflammatory drugs might synergistically contribute to improved survival outcomes.

Our research possesses several notable strengths. Firstly, the diverse analysis methods and extended follow-up duration contribute to the robustness of the results. Secondly, the utilization of sampling weight methods enhances the generalizability of our findings to the American population, to some extent. Notably, our research unveils, for the first time, the potential role of eGFR in mediating the relationship between NLR and mortality. Methodologically, the results of the study remained consistent even after meticulous adjustments for multiple demographic and disease-related variables.

However, this study has the following limitations. Firstly, the NHANES database lacks detailed information on hypertension grading, hindering a more nuanced analysis of the relationship between NLR and mortality across different hypertension grades. Secondly, the scope of adjusted confounding factors in our analysis may not be exhaustive, allowing for potential confounders that could influence the relationship between NLR and mortality. Finally, the results of this study were taken from hypertensive patients in the United States, further investigation is essential to confirm the applicability of the conclusion to hypertensive populations in other countries.

## Conclusion

Our findings indicate a significant and independent association between elevated NLR and the risk of all-cause and cardiovascular mortality in hypertensive patients in the United States. Notably, NLR exhibits a robust predictive capability for short and long-term mortality. Furthermore, eGFR emerged as a mediating factor in the relationship between NLR and mortality. These results suggest that NLR could serve as a simple and convenient tool for identifying high-risk patients and guiding targeted interventions. Further prospective studies are needed to validate these findings and understand the underlying mechanisms.

### Supplementary Information

**Additional file 1: Figure S1.** The flow chart of participants in the current study. **Figure S2.** The cutoff point was calculated using the maximally selected rank statistics based on the ‘maxstat’ package. **Figure S3.** The predictive ability of neutrophil and lymphocyte alone for all‑cause and cardiovascular mortality in patients with hypertension. **Table S1.** Effect of NLR level on cardiovascular mortality: adjusted hazard ratios from segmented cox regression analysis.

## Data Availability

The data used for these analyses are all publicly available at online (https://www.cdc.gov/nchs/nhanes/index.htm).
