# Supplementary material for: The neutrophil-to-lymphocyte ratio is associated with all-cause and cardiovascular mortality among individuals with hypertension
Source: Cardiovasc Diabetol. 2024 Apr 2;23:117. doi: 10.1186/s12933-024-02191-5 (PMC10985955; doi:10.1186/s12933-024-02191-5)
Supplement: Supplementary file 1 — Additional file 1: Figure S1. The flow chart of participants in the current study. Figure S2. The cutoff point was calculated using the maximally selected rank statistics based on the ‘maxstat’ package. Figure S3. The predictive ability of neutrophil and lymphocyte alone for all‑cause and cardiovascular mortality in patients with hypertension. Table S1. Effect of NLR level on cardiovascular mortality: adjusted hazard ratios from segmented cox regression analysis. [file 12933_2024_2191_MOESM1_ESM.docx]

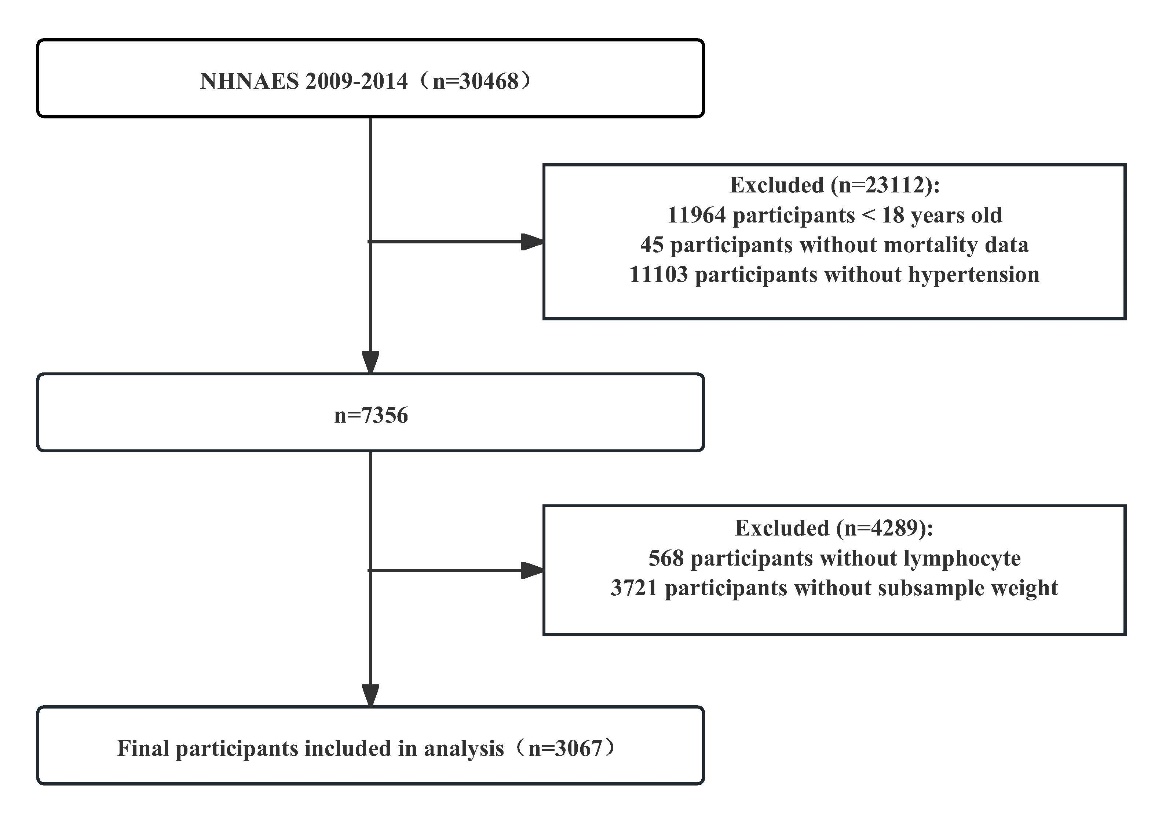


Figure S1. The flow chart of participants in the current study


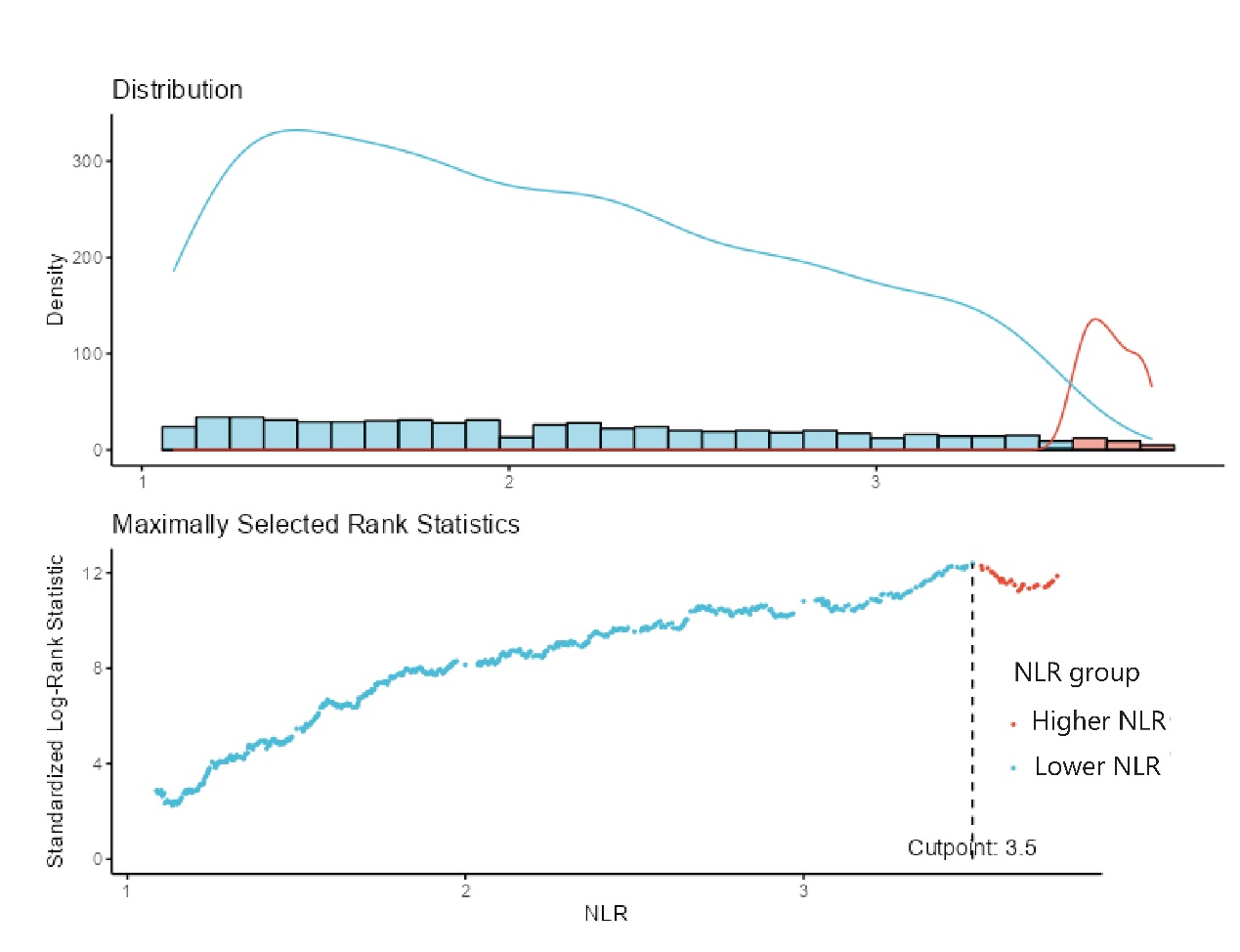


Figure S2. The cutoff point was calculated using the maximally selected rank statistics based on the ‘maxstat’ package.


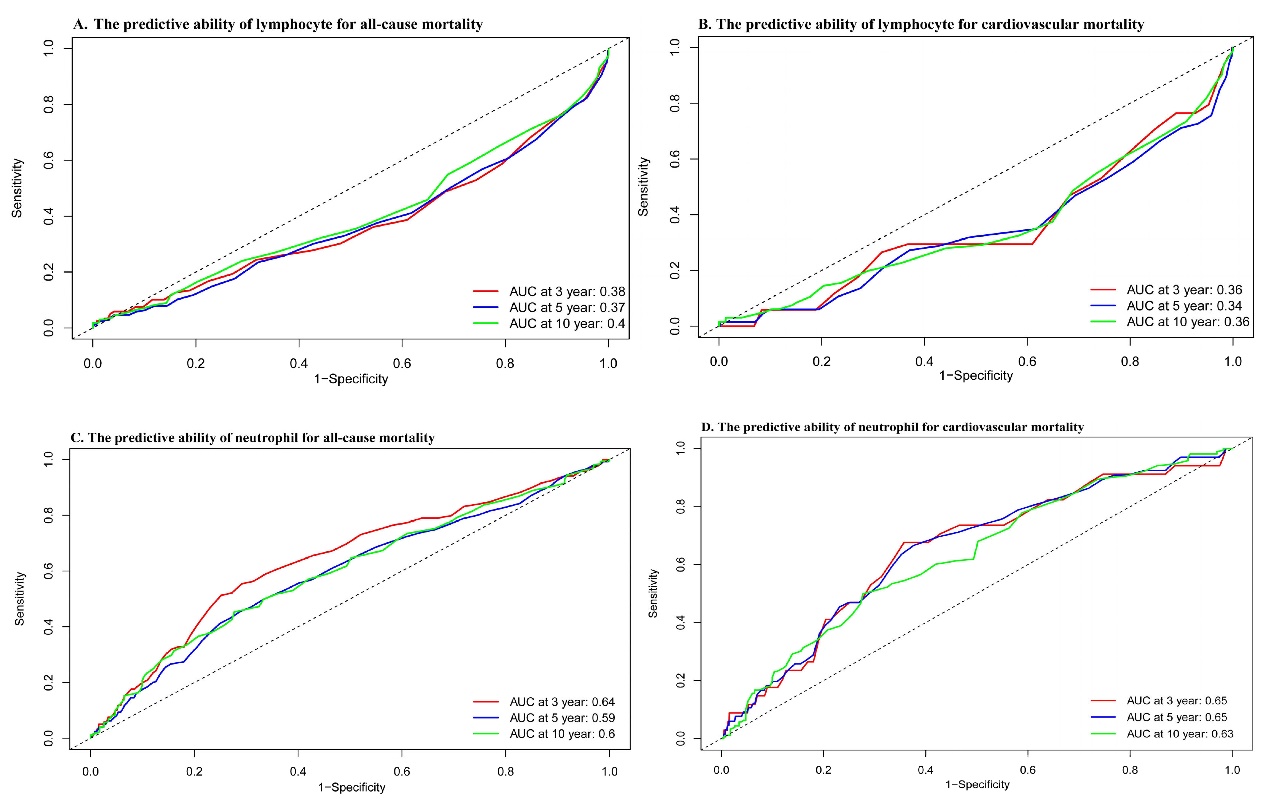


Figure S3. The predictive ability of neutrophil and lymphocyte alone for all‑cause and cardiovascular mortality in patients with hypertension

Table S1. Effect of NLR level on cardiovascular mortality: adjusted hazard ratios from segmented cox regression analysis

| Inflection point | Adjusted HR (95% CI) | P-value |
| --- | --- | --- |
| <2.3 | 2.1(1.09, 4.05) | 0.027 |
| ≥2.3 | 1.12(1.06, 1.18) | <0.001 |
| Log-likelihood ratio | <0.001 |  |

HR was adjusted for age, sex, race, BMI, smoking status, education level, diabetes, history of CVD, HDL, LDL, TG, TC, HbA1c, and the eGFR.
